# Associations between the prevalence of influenza vaccination and patient’s knowledge about antibiotics: A cross-sectional study in the framework of the APRES-project in Austria

**DOI:** 10.1186/s12889-015-2297-x

**Published:** 2015-09-29

**Authors:** Kathryn Hoffmann, Evelien ME van Bijnen, Aaron George, Ruth Kutalek, Elena Jirovsky, Silvia Wojczewski, Manfred Maier

**Affiliations:** Department of General Practice and Family Medicine, Centre for Public Health, Medical University of Vienna, Kinderspitalgasse 15/1st floor, 1090 Vienna, Austria; Netherlands Institute for Health Services Research (NIVEL), Utrecht, The Netherlands; Department of Community and Family Medicine, Duke Medical Center, Durham, NC USA

**Keywords:** Influenza vaccination prevalence, Knowledge about antibiotics, Demographic factors, Primary health care, Austria

## Abstract

**Background:**

This study aimed to identify associations between GP patient’s knowledge about the spectrum of effectiveness of antibiotics and the probability of vaccination against influenza. The underlying hypothesis was that individuals with an understanding that antibiotics are ineffective against viruses, common colds, and flu were more likely to be vaccinated than persons lacking this knowledge.

**Methods:**

This cross-sectional study was conducted within the context of the European APRES project in Austria. Between November 2010 and July 2011, patients were recruited from GP practices to complete questionnaires about their knowledge about antibiotics and their influenza vaccination status. Statistical analyses included subgroup analyses and logistic regression models.

**Results:**

Data of 3224 patients was analyzed, demonstrating that patients with better knowledge concerning antibiotics had a significantly higher likelihood of being vaccinated (OR 1.35, CI 95 % 1.18–1.54). While the overall vaccination rate was low (18.6 % in 2009/2010 and 14.0 % in 2010/2011), elderly compared to younger adults (OR 0.06 CI 95 % 0.03–0.13) and healthcare workers (OR 2.24, CI 95 % 1.42–3.54) demonstrated higher likelihood of vaccination. Additionally, female GPs had significantly more vaccinated patients than male GPs (OR 2.90, CI 95 % 1.32–6.40).

**Discussion:**

There has been little prior study on the association between a patient’s knowledge of the effectiveness spectrum of antibiotics and influenza vaccination status. Given the public health imperative to increase annual prevalence of influenza vaccination, understanding this educational gap can improve specificity in counseling as well as vaccination rates. Ultimately, we found that those with a better knowledge on about antibiotics had a significantly higher likelihood of being vaccinated.

**Conclusions:**

The results of this study demonstrate that vaccination prevalence is associated with patient’s knowledge about antibiotics. It can be concluded that one strategy to improve the overall low vaccination rates for seasonal influenza in Austria would be, particularly for male GPs, to have a specific discussion with patients about these circumstances by focusing on younger patients. Further, public health efforts could supplement in-office strategies to improve this area of health literacy.

**Electronic supplementary material:**

The online version of this article (doi:10.1186/s12889-015-2297-x) contains supplementary material, which is available to authorized users.

## Background

Influenza is a highly infectious viral disease, presenting in one of three basic antigen types A, B or C. The virus can cause moderate to severe illness, with dangerous complications such as secondary bacterial pneumonia, myocarditis or worsening of existing chronic pulmonary or cardiopulmonary diseases [1]. The worldwide burden of influenza is estimated to include approximately one billion cases per year, with three to five million cases of severe illness, and 250,000 to 500,000 influenza-associated deaths [1]. Persons at highest risk are those aged 65 years and older and children under five years; however, influenza affects all age groups [2].

The new generation of influenza vaccines offers an option to protect individuals against influenza [3] but the prevalence of vaccinated persons differs highly within European countries: from 1 % of the elderly population in Estonia to 82 % in the Netherlands [4]. Among healthcare workers, vaccination rates varied from 12 % in Norway to 98 % in Romania in the 2008/2009 flu season [4–7]. The reasons for this variety are manifold: first, official recommendations and funding schemes for flu vaccination differ [4]. Second, various misconceptions including that the influenza vaccination can cause the flu [8], or that upper respiratory symptoms occurring post-vaccination are indicative that the vaccine itself is ineffective, seem to account for much of the variation in vaccinations rates [8, 9]. A recent Cochrane report showed that while the effectiveness of the vaccine remains only modest, vaccination is correlated with a reduced risk of hospitalization for influenza or pneumonia, as well as for respiratory or cardiac diseases, and that there is ultimately reduction in all-cause mortality [10]. Therefore, immunization against influenza remains the most important public health goal to control seasonal, epidemic, and pandemic influenza [4, 6]. Increasing vaccination rates could be achieved by increasing the health literacy of the population regarding influenza and treatment options [7, 10–13], thereby debunking myths about flu vaccination [13]. Moreover, many patients carry the incorrect assumption that antibiotics are effective against influenza [14, 15].

The current study aimed to identify associations between the knowledge about the spectrum of effectiveness of antibiotics, as well as patients’ and general practitioners’ (GPs) demographics and the probability of being vaccinated against seasonal influenza. The underlying hypothesis was that individuals with an understanding that antibiotics are ineffective against viruses, common colds, and flu were more likely to be vaccinated than persons without this knowledge.

## Methods

### Design

Within the framework of the European APRES (Appropriateness of Prescribing Antibiotics in Primary Care in Europe) project, which had the aim to assess the appropriateness of prescribing antibiotics in primary care in nine European countries, and was described previously in detail [15–18], the present cross-sectional study took place in Austria between November 2010 and July 2011. It was the aim that two questionnaires be completed by 4000 patients from 20 GP practices across each federal state. The study design and analysis was in accordance with the STROBE statement for cross-sectional studies (http://www.strobe-statement.org/index.php?id=available-checklists).

#### Recruitment of study participants in Austria

A purposefully selected stratified sample of 20 GPs from within the GP research network of the Department of General Practice at the Medical University of Vienna, with fair representation of the national GP population according to sex, age and geographical location, were recruited [19]. Each of the 20 GPs attempted to recruit 200 consecutive patients aged four years and older. Additionally, the demographic background of the GPs was identified.

Inclusion and exclusion criteria for the patients were the same as in the APRES project and have been published elsewhere [15, 17]. Important for this analysis were the criteria that the patients’ consultation was due to a non-infectious disease and that patients were not allowed to have taken antibiotics in the three months prior to the study. This decreased the risk of patients having any recent exposure to antibiotics and the likelihood that they would have a different level of knowledge about antibiotics. Beyond the overall APRES inclusion and exclusion criteria, an additional exclusion criterion was added for this analysis: patients were required to be 16 years of age or older.

The participating patients completed two questionnaires, one relating to socio-demographic data and seasonal influenza vaccination status, and a second with questions about their knowledge regarding antibiotics.

Additionally, each participant completed a written informed consent form before participation. If the participant was younger than 18 years of age, the parent and adolescent each completed written informed consent forms separately.

#### Questionnaires

The questions assessed included flu vaccination status, sex, age, educational level, country of origin, location of residence, and profession. Age was clustered into four groups: age 16–24 years, 25–44 years, 45–64 years, and 65 years and older. Highest educational level was surveyed in three categories: primary, secondary and tertiary education. The country of origin was assessed by asking “What is your country of origin?”. The variable was grouped into four clusters: Austria, European Union 15 (EU 15) countries including European Free Trade Association (EFTA) countries (EU15+), new EU 28 countries (EU28), and all other countries. To have a better statistical power for the subgroups of this item location of residence was dichotomized into urban areas (big and intermediate cities) and rural areas (small cities, villages and countryside) by means of the European Degree of Urbanization (DEGRUBA) classification [20]. Profession was assessed by asking “Do you work in any of the following occupational fields?” with the answer categories “healthcare”, “livestock farming”, “kindergarten teacher/ (day-) nanny”, “others” or “don’t know”. All socio-demographic factors were defined as demographic control variables.

The seasonal influenza vaccination status was defined as the dependent variable. It was assessed with the two questions “Did you have an influenza vaccination in the winter 2009/2010?” and “Did you have an influenza vaccination in the winter 2010/2011?” with the answer categories “yes”, “no” and “don’t know”. The answer categories were dichotomized into “yes”, versus all other answer options to be able to calculate logistic regression models which was important to be able to answer our hypothesis. For the logistic regression model an additional aggregated variable named “positive two year vaccination status” was constructed.

For the independent variable, knowledge about antibiotics, three related questions were extracted from the knowledge questionnaire. These three questions were identical to those used in the Eurobarometer 2013 report on antimicrobial resistances [14]:

- Question 1: Do antibiotics kill viruses?
- Question 2: Are antibiotics effective against colds and flu?
- Question 3: Does the unnecessary use of antibiotics make them ineffective?

For each question, one of three possible answers could be marked: “yes”, “no” and “don’t know”. Later, these variables were dichotomized into the categories “correctly answered”, versus all other possibilities (false answer, don’t know or not answered at all) to have a better statistical power for the subgroups of this item. Finally, a sum variable “antibiotic knowledge” was built, with one point assigned for each correct answer and total values for all three questions ranging from zero to three points. Furthermore, gender and years of practice (10 years and more or less) of the GPs were taken into account.

### Data analyses

The knowledge score and the answers to the single knowledge questions, as well as all socio-demographic data of the patients and GPs, were related to the influenza vaccination status for the winter seasons 2009/2010, 2010/2011 and for both years. This was done using descriptive statistical methods to be able to present absolute and relative frequencies for all mentioned items by means of cross-tabs and statistical tests: the ANOVA one-way including the Post-Hoc Tukey test for the scale variable knowledge score as well as the Chi-Square independency test and additionally the two-proportional z-test including the Bonferroni method for multiple testing for all other categorical variables. Next, knowledge score and vaccination status was compared between the different GP practices to check for possible group effects. Finally, multi-level logistic regression models were conducted. In the first crude regression model the odds ratio (OR) for the antibiotic knowledge score in relation to a positive two year influenza vaccination status was calculated to see if a crude association was present. In the second regression model the knowledge score as well as all socio-demographic factors of the patients were included simultaneously to adjust for other relevant factors and confounders. In the third regression model, sex and age of GPs were included, as well as an adjustment for the GP practice codes to account for any possible inter-practice effect. This was performed by building a dichotomous dummy variable for each GP practice, which was included in the model.

The significance level for all calculations was *p* < 0.05 and the confidence interval 95 %. SPSS Statistics version 22.0 was used for the statistical analyses.

### Ethical considerations

The study was approved by the Ethics Committee of the Medical University Vienna (EC # 568/2010).

## Results

### Sample

The participating 20 GPs recruited a total of 3224 patients aged 16 years and older who completed the two questionnaires. The distribution of the socio-demographic factors of the patients’ sample as well as the proportions of correct answers to the three antibiotics knowledge questions is shown in Table 1. The influenza vaccination status was assessed for each of two winter seasons, with the rate of vaccination demonstrating values of 18.6 % for 2009/2010, 14.0 % for 2010/2011 and 12.1 % for patients that were vaccinated in both seasons. The mean knowledge score about antibiotics was 1.35 points out of a total possible score of three points.

**Table 1:** Influenza vaccination frequencies in relation to AB knowledge and socio-demographic data

			Flu vaccination 2009/2010		Flu vaccination 2010/2011		Both years flu vaccination	
Variable	Sub-variable	All	Yes	No/Don’t know	Yes	No/Don’t know	Yes	No/Don’t know
		Mean (SD)	Mean (SD)	Mean (SD)	Mean (SD)	Mean (SD)	Mean (SD)	Mean (SD)
AB-knowledge-Score		1.35 (0.99)	1.49 (1.06)	1.32 (0.98)	1.51 (1.06)	1.34 (0.98)	1.54 (1.07)	1.33 (0.98)
*p*			0.001		0.002		<0.001	
		% (*n*)	% (*n*)	% (*n*)	% (*n*)	% (*n*)	% (*n*)	% (*n*)
All		100.0 (3224)	18.6 (593)	81.4 (2598)	14.0 (435)	86.0 (2675)	12.1 (383)	87.9 (2780)
Question 1^d^	Correct	28.0 (894)	22.6 (201)_a_	77.4 (689)_a_	16.8 (148)_a_	83.2 (731)_a_	14.8 (131)_a_	85.2 (754)_a_
	Not correct	72.0 (2298)	17.0 (386)_b_	83.0 (1886)_b_	12.7 (281)_b_	87.3 (1923)_b_	10.9 (246)_b_	89.1 (2003)_b_
*p*			<0.001		0.003		0.003	
Question 2^e^	Correct	32.7 (1043)	21.8 (226)_a_	78.2 (812)_a_	16.4 (167)_a_	83.6 (850)_a_	15.1 (156)_a_	84.9 (875)_a_
	Not correct	67.3 (2149)	17.0 (361)_b_	83.0 (1763)_b_	12.7 (262)_b_	87.3 (1804)_b_	10.5 (221)_b_	89.5 (1882)_b_
*p*			0.001		0.005		<0.001	
Question 3^f^	Correct	74.2 (2368)	18.9 (445)_a_	81.1 (1906)_a_	14.4 (333)_a_	85.6 (1978)_a_	12.6 (295)_a_	87.4 (2039)_a_
	Not correct	25.8 (824)	17.5 (142)_a_	82.5 (669)_a_	12.4 (96)_a_	87.6 (676)_a_	10.3 (82)_a_	89.8 (718)_a_
*p*			0.370		0.170		0.073	
Sex	Female	56.6 (1801)	18.9 (337)_a_	81.1 (1448)_a_	13.5 (235)_a_	86.5 (1506)_a_	11.9 (211)_a_	88.1 (1560)_a_
	Male	43.4 (1382)	18.1 (247)_a_	81.9 (1121)_a_	14.3 (190)_a_	85.7 (1141)_a_	12.1 (164)_a_	87.9 (1189)_a_
*p*			0.555		0.562		0.868	
Age	16–24	9.0 (290)	8.4 (24)_a_	91.6 (263)_a_	4.6 (13)_a_	95.4 (272)_a_	2.8 (8)_a_	97.2 (279)_a_
	25–44	30.6 (985)	10.7 (105)_a_	89.3 (874)_a_	6.6 (64)_a_	93.4 (905)_a_	5.4 (53)_a_	94.6 (920)_a_
	45–64	36.8 (1185)	17.1 (201)_b_	82.9 (975)_b_	11.9 (135)_b_	88.1 (1004)_b_	10.3 (120)_b_	89.7 (1046)_b_
	65+	23.7 (764)	35.1 (263)_c_	64.9 (486)_c_	31.1 (223)_c_	68.9 (494)_c_	27.4 (202)_c_	72.6 (535)_c_
*p*			<0.001		<0.001		<0.001	
Educational level	Primary	48.6 (1539)	17.6 (267)_a_	82.4 (1254)_a_	13.4 (198)_a_	86.6 (1276)_a_	11.2 (169)_a_	88.8 (1337)_a_
	Secondary	37.5 (1185)	18.4 (217)_a_	81.6 (961)_a_	13.6 (157)_a_	86.4 (995)_a_	12.1 (141)_a_	87.9 (1025)_a_
	Tertiary	13.9 (440)	22.5 (98)_a_	77.5 (338)_a_	16.3 (70)_a_	83.7 (360)_a_	14.5 (63)_a_	85.5 (372)_a_
*p*			0.065		0.307		0.183	
Country of origin	Austria	85.9 (2729)	18.9 (511)_a_	81.1 (2191)_a_	14.6 (384)_a_	85.4 (2253)_a_	12.5 (336)_a_	87.5 (2346)_a_
	EU 15+	3.2 (102)	22.8 (23)_a_	77.2 (78)_a_	13.1 (13)_a,b_	86.9 (86)_a,b_	12.1 (12)_a_	87.9 (87)_a_
	New EU 28	2.9 (93)	16.1 (15)_a_	83.9 (78)_a_	11.0 (10)_a,b_	89.0 (81)_a,b_	9.8 (9)_a_	90.2 (83)_a_
	Others	8.0 (253)	13.9 (35)_a_	86.1 (216)_a_	7.9 (19)_b_	92.1 (222)_b_	6.9 (17)_a_	93.1 (229)_a_
*p*			0.150		0.030		0.054	
Location of residence	Urban	44.7 (1440)	22.8 (324)_a_	77.2 (1098)_a_	18.2 (253)_a_	81.8 (1140)_a_	15.7 (220)_a_	84.3 (1185)_a_
	Rural	55.3 (1784)	15.2 (269)_b_	84.8 (1500)_b_	10.6 (182)_b_	89.4 (1535)_b_	9.3 (163)_b_	90.7 (1595)_b_
*p*			<0.001		<0.001		<0.001	
Job	Health care	6.0 (194)	26.8 (52)_b_	73.2 (142)_b_	18.2 (35)_a_	81.8 (157)_a_	15.0 (29)_a_	85.0 (164)_a_
	Livestock farming	2.9 (93)	9.7 (9)_c_	90.3 (84)_c_	6.7 (6)_a_	93.3 (83)_a_	6.5 (6)_a_	93.5 (87)_a_
	Kindergarten teacher	1.9 (60)	10.0 (6)_a,b,c_	90.0 (54)_a,b,c_	3.5 (2)_a_	96.5 (55)_a_	3.4 (2)_a_	96.6 (56)_a_
	Others	80.6 (2598)	17.9 (461)_a,c_	82.1 (2113)_a,c_	13.8 (347)_a_	86.2 (2175)_a_	11.9 (305)_a_	88.1 (2251)_a_
*p*	Not known	8.7 (279)	24.1 (65)_a,b_	75.9 (205)_a,b_	18.0 (45)_a_	82.0 (205)_a_	15.6 (41)_a_	84.4 (222)_a_
			<0.001		0.002		0.017	

^a, b, c^The minuscule letters behind the percentages (_a, b, c_) represent a subset of the variable category which is not significantly different at a significance level of *p* < 0.05 if it is the same miniscule for the same category (column)

^d^Do antibiotics kill viruses?

^e^Are antibiotics effective against colds and flu?

^f^Does unnecessary use of antibiotics make them ineffective?

The GPs were on average 51.6 years old (SD 4.893, range 37–59). Among participating GPs, 30.0 % were female (*n* = 6), 70.0 % male (*n* = 14), and 90.4 % (*n* = 18) of all GPs had practiced for 10 years or more.

### Patients with better knowledge about antibiotics demonstrated increased likelihood to be vaccinated against influenza

The cross-tab in Table 1 depicts that patients with a better knowledge about antibiotics were significantly more likely to be vaccinated against influenza than those with inferior knowledge (knowledge score 1.54 (SD1.07) vs. 1.33 (SD 0.98); *p* < 0.001). Particularly, patients who demonstrated an accurate knowledge about the spectrum of effectiveness of antibiotics were found to be more frequently vaccinated in each season as well as both seasons together (14.8 % vs. 10.9 %; *p* = 0.003; Table 1). We found that persons aged 65 years and older were more frequently vaccinated, as it was the case for those living in urban areas. Furthermore, occupation was found to have an association with the influenza vaccination status: healthcare workers were most frequently (26.8 %) and kindergarten teachers least frequently (10.0 %) vaccinated (Table 1).

**Fig. 1:**
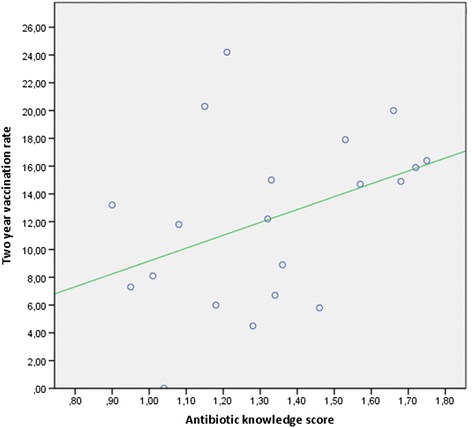
Correlation between average vaccination rates and knowledge scores for the single GP practices

Table 2 depicts the differences between the GP groups, showing high variability among GP practices regarding the influenza vaccination rate for the two seasons, with ranges from zero to 24.2 %. This was also observed in the relationship to antibiotic knowledge score, with ranges from 0.90 points to 1.75 points. A small, but not significant correlation between the mean antibiotic knowledge score and the average two year vaccination rate could be found (correlation coefficient 0.391; p 0.088) (Fig. 1).

**Table 2:** Vaccination rate and AB knowledge score per GP practice

GP practice	Vaccination rate for 2 years	AB-knowledge score
	% (*n*)	Mean (CI 95 %)
1 (*n* = 199)	8.9 (17)_a,b,c,d_	1.36 (1.22–1.50)
2 (*n* = 111)	11.8 (13)_a,b,c,d_	1.08 (0.91–1.24)
3 (*n* = 199)	12.2 (24)_a,b,c,d_	1.32 (1.18–1.45)
4 (*n* = 213)	8.1 (17) _a,b,c,d_	1.01 (0.90–1.13)
5 (*n* = 185)	13.2 (24)_a,b,c,d_	0.90 (0.77–1.00)
6 (*n* = 62)	24.2 (15)_c,d_	1.21 (0.96–1.46)
7 (*n* = 168)	16.4 (27)_a,b,c,d_	1.75 (1.60–1.90)
8 (*n* = 193)	15.0 (29)_a,b,c,d_	1.33 (1.20–1.46)
9 (*n* = 195)	7.3 (14)_a,b_	0.95 (0.83–1.08)
10 (*n* = 150)	15.9 (23)_a,b,c,d_	1.72 (1.55–1.89)
11 (*n* = 210)	14,9 (31)_a,b,c,d_	1.68 (1.55–1.82)
12 (*n* = 194)	14.7 (28)_a,b,c,d_	1.57 (1.43–1.71)
13 (*n* = 181)	17.9 (32)_a,b,c,d_	1.53 (1.38–1.67)
14 (*n* = 196)	20.0 (37)_b,d_	1.66 (1.52–1.80)
15 (*n* = 113)	4.5 (5)_a_	1.28 (1.09–1.47)
16 (*n* = 183)	6.7 (12)_a_	1.34 (1.15–1.46)
17 (*n* = 26)	0	1.04 (0.69–1.39)
18 (*n* = 68)	20.3 (13)_a,b,c,d_	1.15 (0.92–1.38)
19 (*n* = 193)	5.8 (11)_a_	1.46 (1.31–1.61)
20 (*n* = 185)	6.0 (11)_a_	1.18 (1.04–1.32)
	Chi-Square (Exact) *p* < 0.001	ANOVA *p* < 0.001; homogeneity of variances *p* < 0.001

^a, b, c^The minuscule letters behind the percentages (_a, b, c, d_) represent a subset of the variable category which is not significantly different at a significance level of *p* < 0.05 if it is the same miniscule for the same category

The first crude regression model demonstrates that the antibiotic knowledge score is significantly and positively associated with the likelihood of being vaccinated (OR 1.24 CI 95 % 1.11–1.38). This association remained significant for the two adjusted models (model two and three) that included both multiple patient as well as GP and GP practice related factors (Table 3).

**Table 3:** Regression models for the association of the AB-knowledge score with the likelihood to be vaccinated in both years surveyed (*n* = 3051)

Variable	Sub-variable	Model I (crude)		Model II (adjusted)		Model III (adjusted)^a^	
		OR (95 %)	*p*	OR (95 %)	*p*	OR (95 %)	*p*
AB-Knowledge score		1.24 (1.11–1.38)	<0.001	1.29 (1.14–1.47)	<0.001	1.35 (1.18–1.54)	<0.001
Sex	Female			0.87 (0.69–1.11)	0.259	0.85 (0.67–1.08)	0.191
	male			1.0		1.0	
Age	16–24			0.06 (0.03–0.13)	<0.001	0.05 (0.02–0.10)	<0.001
	25–44			0.13 (0.09–1.18)	<0.001	0.11 (0.08–0.16)	<0.001
	45–64			0.27 (0.20–0.35)	<0.001	0.24 (0.18–0.32)	<0.001
	65+			1.0		1.0	
Educational level	Primary			1.0		1.0	
	Secondary			1.12 (0.86–1.47)	0.402	1.13 (0.85–1.49)	0.398
	Tertiary			1.28 (0.88–1.86)	0.196	1.32 (0.89–1.96)	0.168
Country of origin	Austria			1.0		1.0	
	EU 15+			0.65 (0.32–1.30)	0.219	0.67 (0.33–1.36)	0.671
	New EU 28			0.86 (0.41–1.83)	0.699	0.89 (0.41–1.91)	0.763
	Others			0.63 (0.37–1.07)	0.086	0.68 (0.39–1.17)	0.166
Location of residence	Urban			1.62 (1.26–2.08)	<0.001	2.13 (1.0–4.63)	0.050
	Rural			1.0		1.0	
Job	Health care			2.24 (1.42–3.54)	0.001	2.28 (1.43–3.64)	0.001
	Livestock farming			0.71 (0.30–1.68)	0.707	0.52 (0.21–1.28)	0.152
	Kindergarten teacher			0.25 (0.03–1.86)	0.176	0.27 (0.04–2.00)	0.197
	Not known			1.33 (0.89–1.99)	0.163	1.59 (1.04–2.44)	0.033
	Others			1.0		1.0	
GP gender	Female					2.90 (1.32–6.40)	0.008
	Male					1.0	
GP Experience	<10 years					0.64 (0.21–1.14)	0.439
	>10 years					1.0	
Nagelkerkes R^2^		0.011		0.168		0.206	

^a^Model III additionally adjusted for GP practice code

The second model additionally showed that an age under 65 years demonstrated a significantly decreased probability of having been vaccinated against influenza (OR 0.06 CI 95 % 0.03–0.13). In contrast, living in an urban area or working in the health care sector significantly increased the likelihood of vaccination (Table 3).

The third model, with inclusions of GP factors, illustrates that these factors continued to demonstrate significant associations with antibiotic knowledge (OR 1.35 CI 95 % 1.18–1.54) and that the inclusion of the GP practice codes eliminated the significance of those living in urban areas. Moreover, female sex of the GP was significantly associated with a higher probability of patients that had been vaccinated (OR 2.90 CI 95 % 1.32–6.40).

## Discussion

Health literacy has been shown to have an impact on vaccination status [2, 9–13], and inaccurate beliefs about antibiotics have been recognized [14, 15]. However, no prior study was found that addressed the association between a patient’s knowledge of the effectiveness spectrum of antibiotics and influenza vaccination status. Given the public health imperative to increase annual prevalence of influenza vaccination [21], understanding this educational gap can improve specificity in counseling by physicians and boost vaccination rates.

We identified an association between knowledge concerning the effectiveness spectrum of antibiotics and the probability of being vaccinated against influenza. Persons with a better knowledge had a significantly higher likelihood of being vaccinated in all regression models, with an OR of 1.35 in the fully adjusted model which was adjusted for patient and GP factors. Specifically, the two questions regarding the effectiveness spectrum of antibiotics showed a significant association with a positive vaccination status. The overall knowledge about antibiotics remains low in Austria [14, 15], as is the overall level of health literacy [22]. Less than one third of the Austrian population have been shown to recognize that antibiotics do not kill viruses, and this is also true for nearly half of those with tertiary education [15]. The reason that the adjusted results did not show an association between educational level and the seasonal influenza vaccination status could be due to a bias: educational status might be already included as a relevant factor in the antibiotics knowledge variable which has been shown as having an association with the educational level in a previous publication [15]. In the Additional file 1 it becomes obvious that tertiary educational level increased the likelihood of being vaccinated (crude model), however, this association vanished when including the antibiotic knowledge variable (adjusted models in Table 3). The European Health Literacy Survey, which has measured how people access, understand, appraise and apply information to make decisions in health care, showed that Austria ranked in the lower field regarding those literacy items [22]. It could be speculated that improvements in health literacy, particularly regarding the spectrum of antibiotic effectiveness, would result in an improvement in vaccination status. GPs could specifically address the topic of non-effectiveness of antibiotics for the treatment of the flu, or colds without superinfections, at the time of discussion of influenza vaccination. This clinical guidance could include debunking inaccuracies, such as the assumption that the influenza vaccination can cause flu or that it is not effective following a viral upper respiratory infection. Furthermore, professional counseling on the differences between bacterial and viral infections may encourage more patients to seek vaccination.

We identified the highest vaccination rates during the season 2009/2010 with 18.6 % of patients receiving the vaccine. In the season 2010/2011, the rate was only 14.0 %. This may be explained by the recruitment timeframe, which was between November 2010 and July 2011, meaning that patients recruited in November and December 2010 probably were not yet vaccinated. Another explanation could be the decrease in motivation to vaccinate after the flu pandemic in 2009 [23]. The overall low vaccination status is reflected by other studies, in which Austria has been described as one of the three Western European countries with the lowest rate of vaccinations [5, 24, 25]. In the most recent Austrian health literacy survey 2006/2007, about 20 % of participants indicated they were vaccinated against influenza [26]. Another explanation for the overall low influenza vaccination prevalence compared to other European countries could be the manner in which influenza vaccination is organized in Austria [4, 5]. Although influenza vaccination is officially recommended, vaccination remains voluntary and most Austrians have to pay for the vaccination out of their pocket. This is in contrast to, for example, the Netherlands, where elderly populations have funding for vaccination and the rate of vaccination is among 80 % [4].

Overall, older age, living in an urban area, and being a healthcare worker each demonstrated an increased likelihood of vaccination in our analysis. Among healthcare workers, the vaccination rate is higher than in the average sample, but with only 26.8 % in the season 2009/2010. This phenomenon has been observed elsewhere, as a recent Spanish study showed rates of healthcare workers approaching 31 %, and a decreasing trend after the year 2009 [23]. Another Spanish study showed that vaccination was higher in healthcare workers who recognized vaccination as effective and those worried about being infected or infecting patients leading to the assumption that improving health literacy even in healthcare workers could have a large effect in improving vaccination rates [27]. The vaccination rate in Austria, in contrast, was particularly low in kindergarten teachers. This result is concerning, as these persons can infect many children once they carry the virus [28]. However, the low numbers could also be a result of the smaller subgroup sample.

The highest prevalence of vaccination was observed in persons aged 65 years and older, with 35.1 % identified in the season 2009/2010. This result is promising as the elderly are a vulnerable group in relation to severe illnesses caused by the influenza virus; however, still far away from the goal of the 10^th^ World Health Assembly resolution [21]. Contrary to this, vaccination rates in persons under the age of 24 are very low, with only 8.4 % observed in the season 2009/2010.

The high variability of the vaccination status between the GP practices observed could lead to the assumption that personal engagement of the GP is more important than official recommendations. However, despite this variability, only a small correlation was found between vaccination status and the knowledge score between GP practices. Interestingly, female GPs had a higher likelihood to have vaccinated patients with an OR of 2.90, after adjustment for multiple patients’ demographic factors. The GP sample is small and, therefore, no generalizations could be drawn. Nevertheless, it is still noteworthy that similar results have been observed for other vaccines, such as varicella, where female GPs were found to be more likely to discuss immunization with recommended, non-funded vaccines [29], or HPV vaccination where female GPs in Australia had significantly higher rates of vaccinated patients [30].

The strengths of the present study were the large sample size and the similarity of the sample with the Austrian population with regard to sex, age and educational level [31]. However, there may still be some differences from the general population, because the sample groups were recruited from general practices. Another limitation is the fact that this study is cross-sectional and, therefore, of limited explanatory power. Furthermore, results are based on descriptive and self-reported survey data. In addition, the lack of a question regarding chronic conditions might have resulted in biasing the results, because chronic conditions are recognized as important predictors for being vaccinated against seasonal influenza [27, 32]. Furthermore, the division of the vaccination status variable in “yes” and “no/don’t know” did not consider those patients who probably were vaccinated but could not remember it anymore which could have led to an underestimation of patients vaccinated; however, the detailed analysis shown in the Additional file 2 shows that only few patients marked “don’t know”. In addition, it became obvious that, although patients that marked “don’t know” had a lower antibiotic knowledge score, these persons did not have a statistical effect on the overall regression model results presented in Table 3 (Additional files 2 and 3). Other limitations were the voluntary recruitment strategy of GPs and patients and the fact that the questionnaire was available only in German. It may be speculated that more GPs and patients interested in the topic of antibiotic resistance participated in the study, which might have over-estimated the real knowledge about antibiotics.

## Conclusion

Austria, as well as many other European countries, still has the need for massive improvements in influenza vaccination rates. We observed that in any given flu season only as much as 18.6 %, and as little as 14 %, of the population were vaccinated. Recognizing the public health imperative to reduce the spread of flu and decrease potential complications, global strategies must be implemented to increase vaccination rates. The results of this study demonstrate that vaccination prevalence has associations with patient’s knowledge about the effectiveness spectrum of antibiotics. Those patients that had an increased understanding of antibiotic uses were more likely to be vaccinated against influenza. One strategy to improve rates of vaccination for influenza would be for physicians to specifically counsel patients concerning antibiotics, flu vaccination and misconceptions about respiratory infections at the time of suggested vaccination. Furthermore, public health and marketing efforts could supplement in-office strategies to improve this area of health literacy, including the funding of the vaccination for vulnerable groups.

## Additional files

Additional file 1:
**Crude results of the logistic regression model for all variables separately regarding associations with a positive seasonal influenza two year vaccination status.** (DOCX 13 kb) Additional file 2:
**Influenza vaccination status in detail (yes, no, don’t know, no/don’t know) by AB knowledge score.** (DOCX 15 kb) Additional file 3:
**Crude regression model for the association of the AB knowledge score with the likelihood to be vaccinated in both years surveyed (only “yes” and “no” answers regarding vaccination status were taken into account, “don’t know” answers were excluded).** (DOCX 11 kb)

## Abbreviations

ANOVA: Analysis of variance
APRES: Project financed by the seventh EU framework program “APRES – The appropriateness of prescribing antibiotics in primary health care in Europe with respect to antibiotic resistance”
DEGRUBA: Degree of Urbanization
EFTA: European Free Trade Association
EU: European Union
Flu: Influenza
GP: General practitioner
HPV: Human papilloma virus
OR: Odds ratio
SD: Standard deviation
STROBE: Stands for an international, collaborative initiative of epidemiologists, methodologists, statisticians, researchers and journal editors involved in the conduct and dissemination of observational studies, with the common aim of STrengthening the Reporting of OBservational studies in Epidemiology

## References

[1] World Health Organization. Topics: immunizations, vaccines and biologicals. 2008. http://www.who.int/topics/influenza/en/. Accessed 7 August 2015.

[2] Nagata JM, Hernandez-Ramos I, Kurup AS, Albrecht D, Vivas-Torrealba C, Franco-Paredes C (2013). Social determinants of health and seasonal influenza vaccination in adults >/=65 years: a systematic review of qualitative and quantitative data. BMC Public Health..

[3] Stephenson I, Gust I, Kieny MP, Pervikov Y (2006). Development and evaluation of influenza pandemic vaccines. Lancet Infect Dis.

[4] Mereckiene J, Cotter S, Nicoll A, Lopalco P, Noori T, Weber J (2014). Seasonal influenza immunisation in Europe. Overview of recommendations and vaccination coverage for three seasons: pre-pandemic (2008/09), pandemic (2009/10) and post-pandemic (2010/11). Euro Surveill.

[5] Influenza H1N1. Analytical Report. In: Flash Eurobarometer 287. European Commission; 2010.

[6] Kieny MP, Costa A, Hombach J, Carrasco P, Pervikov Y, Salisbury D (2006). A global pandemic influenza vaccine action plan. Vaccine.

[7] Thomas RE, Jefferson T, Lasserson TJ (2010). Influenza vaccination for healthcare workers who work with the elderly. Cochrane Database Syst Rev..

[8] Center for Disease Control and Prevention. Misconceptions about seasonal flu and flu vaccines. 2015http://www.cdc.gov/flu/about/qa/misconceptions.htm. Accessed 9 September 2015.

[9] Prior L, Evans MR, Prout H (2011). Talking about colds and flu: the lay diagnosis of two common illnesses among older British people. Soc Sci Med.

[10] Jefferson T, Di Pietrantonj C, Rivetti A, Bawazeer GA, Al-Ansary LA, Ferroni E (2010). Vaccines for preventing influenza in healthy adults. Cochrane Database Syst Rev..

[11] Bonfiglioli R, Vignoli M, Guglielmi D, Depolo M, Violante FS (2013). Getting vaccinated or not getting vaccinated? Different reasons for getting vaccinated against seasonal or pandemic influenza. BMC Public Health..

[12] Jefferson T, Di Pietrantonj C, Al-Ansary LA, Ferroni E, Thorning S, Thomas RE (2010). Vaccines for preventing influenza in the elderly. Cochrane Database Syst Rev..

[13] Nyhan B, Reifler J (2015). Does correcting myths about the flu vaccine work? An experimental evaluation of the effects of corrective information. Vaccine.

[14] Special Eurobarometer Report on Antimicrobial Resistance. European Commission, Directorate-General for Health and Consumers; 2013.

[15] Hoffmann K, Ristl R, Heschl L, Stelzer D, Maier M (2014). Antibiotics and their effects: what do patients know and what is their source of information?. Eur J Public Health.

[16] den Heijer CD, van Bijnen EM, Paget WJ, Pringle M, Goossens H, Bruggeman CA (2013). Prevalence and resistance of commensal Staphylococcus aureus, including meticillin-resistant S aureus, in nine European countries: a cross-sectional study. Lancet Infect Dis.

[17] van Bijnen EM, den Heijer CD, Paget WJ, Stobberingh EE, Verheij RA, Bruggeman CA (2011). The appropriateness of prescribing antibiotics in the community in Europe: study design. BMC Infect Dis..

[18] van Bijnen EM, Paget WJ, den Heijer CD, Stobberingh EE, Bruggeman CA, Schellevis FG (2014). Primary care treatment guidelines for skin infections in Europe: congruence with antimicrobial resistance found in commensal Staphylococcus aureus in the community. BMC Fam Pract..

[19] Austrian Chamber of Physicians. [Niedergelassese Ärzte - Demographiestrukturauswertung]. Closed database. 2011.

[20] Eurostat. The new degree of urbanisation. 2011. http://ec.europa.eu/eurostat/ramon/miscellaneous/index.cfm?TargetUrl=DSP_DEGURBA. Accessed 7 August 2015.

[21] Resolution of the World Health Assembly (WHA 56.19). Prevention and control of influenza pandemics abd annual epidemics. WHA 10th plenary meeting. World Health Organization; 2003.

[22] Maastricht University, the Netherlands. The European Health Literacy Survey (HLS-EU). 2013. http://www.maastrichtuniversity.nl/web/Institutes/FHML/CAPHRI/DepartmentsCAPHRI/InternationalHealth/ResearchINTHEALTH/Projects/HealthLiteracyHLSEU/MeasuringHealthLiteracyInEurope.htm. Accessed 7 August 2015.

[23] Jimenez-Garcia R, Rodriguez-Rieiro C, Hernandez-Barrera V, Carrasco Garrido P, Lopez de Andres A, Esteban-Vasallo MD (2014). Negative trends from 2008/9 to 2011/12 seasons in influenza vaccination coverages among high risk subjects and health care workers in Spain. Vaccine.

[24] Kunze U, Bohm G, Groman E (2013). Influenza vaccination in Austria from 1982 to 2011: a country resistant to influenza prevention and control. Vaccine.

[25] Kunze U, Groman E, Bohm G, Kunze M (2007). Influenza vaccination in Austria, 1982–2003. Wien Med Wochenschr.

[26] Klimont J, Ihle P, Baldaszti E, Kytir J (2008). Sozio-demographische und sozio-ökonomische Determinanten von Gesundheit. Auswertungen der Daten aus der Österreichischen Gesundheitsbefragung 2006/2007.

[27] Castilla J, Martinez-Baz I, Godoy P, Toledo D, Astray J, Garcia S (2013). Trends in influenza vaccine coverage among primary healthcare workers in Spain, 2008–2011. Prev Med.

[28] Lau CH, Springston EE, Sohn MW, Mason I, Gadola E, Damitz M (2012). Hand hygiene instruction decreases illness-related absenteeism in elementary schools: a prospective cohort study. BMC Pediatr..

[29] Marshall H, Ryan P, Roberton D, Beilby J (2009). Varicella immunisation practice: implications for provision of a recommended, non-funded vaccine. J Paediatr Child Health.

[30] Mazza D, Petrovic K, Chakraborty S (2012). HPV vaccination of adult women: an audit of Australian general practitioners. Aust N Z J Obstet Gynaecol.

[31] Statistics Austria. Demographic distribution of the population in Austria 2008–2012. http://www.statistik.at/web_de/statistiken/bevoelkerung/volkszaehlungen_registerzaehlungen_abgestimmte_erwerbsstatistik/bevoelkerung_nach_demographischen_merkmalen/index.html. Accessed 7 August 2015.

[32] Mereckiene J, Cotter S, D’Ancona F, Giambi C, Nicoll A, Levy-Bruhl D (2010). Differences in national influenza vaccination policies across the European Union, Norway and Iceland 2008–2009. Euro Surveill..

