# Supplementary material for: Associations between the prevalence of influenza vaccination and patient’s knowledge about antibiotics: A cross-sectional study in the framework of the APRES-project in Austria
Source: BMC Public Health. 2015 Sep 29;15:981. doi: 10.1186/s12889-015-2297-x (PMC4587920; doi:10.1186/s12889-015-2297-x)
Supplement: Additional file 1: — Crude results of the logistic regression model for all variables separately regarding associations with a positive seasonal influenza two year vaccination status. (DOCX 13 kb) [file 12889_2015_2297_MOESM1_ESM.docx]

**Additional file 1.** Crude results of the logistic regression model for all variables separately regarding associations with a positive seasonal influenza two year vaccination status

| Variable | Sub-variable | Model I  (crude, all variables separately) | |
| --- | --- | --- | --- |
|  |  | OR (95%) | p |
| AB-Knowledge score | | 1.24 (1.11-1.38) | <0.001 |
| Sex | female | 1.05 (0.84-1.30) | 0.680 |
|  | male | 1.0 |  |
| Age | 16-24 | 0.08 (0.04-0.16) | <0.001 |
|  | 25-44 | 0.15 (0.11-0.21) | <0.001 |
|  | 45-64 | 0.30 (0.24-0.39) | <0.001 |
|  | 65+ | 1.0 |  |
| Educational level | Primary | 1.0 |  |
|  | Secondary | 1.09 (0.86-1.38) | 0.486 |
|  | Tertiary | 1.34 (1.01-1.83) | 0.048 |
| Country of origin | Austria | 1.0 |  |
|  | EU 15+ | 0.96 (0.52-1.78) | 0.904 |
|  | New EU 28 | 0.76 (0.38-1.52) | 0.434 |
|  | Others | 0.52 (0.31-0.86) | 0.011 |
| Location of residence | Urban | 1.82 (1.46-2.26) | <0.001 |
|  | Rural | 1.0 |  |
| Job | Health care | 1.31 (0.86-1.97) | 0.206 |
|  | Livestock farming | 0.51 (0.22-1.17) | 0.113 |
|  | Kindergarten teacher | 0.26 (0.06-1.09) | 0.065 |
|  | Not known | 1.36 (0.96-1.94) | 0.086 |
|  | Others | 1.0 |  |
| GP gender | female | 1.47 (1.18-1.83) | 0.001 |
|  | male | 1.0 |  |
| GP Experience | <10 years | 1.24 (0.88-1.75) | 0.226 |
|  | >10 years | 1.0 |  |
