# Supplementary material for: Associations between the prevalence of influenza vaccination and patient’s knowledge about antibiotics: A cross-sectional study in the framework of the APRES-project in Austria
Source: BMC Public Health. 2015 Sep 29;15:981. doi: 10.1186/s12889-015-2297-x (PMC4587920; doi:10.1186/s12889-015-2297-x)
Supplement: Additional file 2: — Influenza vaccination status in detail (yes, no, don’t know, no/don’t know) by AB knowledge score. (DOCX 15 kb) [file 12889_2015_2297_MOESM2_ESM.docx]

Additional file 2. Influenza vaccination status in detail (yes, no, don´t know, no/don´t know) by AB knowledge score

| **Variable** | **Flu vaccination 2009/2010** | | | | **Flu vaccination 2010/2011** | | | | **Both years flu vaccination** | | |
| --- | --- | --- | --- | --- | --- | --- | --- | --- | --- | --- | --- |
|  | Yes | No | Don´t know | No/Don´t know | Yes | No | Don´t know | No/Don´t know | Yes | No | No/Don´t know |
|  | % (n) | % (n) | % (n) | % (n) | % (n) | % (n) | % (n) | % (n) | % (n) | % (n) | % (n) |
| All | 18.6 (593) | 80.2 (2560) | 1.4 (38) | 81.4 (2598) | 14.0 (435) | 84.8 (2638) | 1.2 (37) | 86.0 (2675) | 12.1 (383) | 83.8 (2652) | 87.9 (2780) |
|  | Mean (SD) | Mean (SD) | Mean (SD) | Mean (SD) | Mean (SD) | Mean (SD) | Mean (SD) | Mean (SD) | Mean (SD) | Mean (SD) | Mean (SD) |
| AB-knowledge score | 1.49 (1.06) | 1.33 (0.98) | 0.84 (0.86) | 1.32 (0.98) | 1.51 (1.06) | 1.35 (0.98) | 0.97 (0.87) | 1.34 (0.98) | 1.54 (1.07) | 1.35 (0.98) | 1.33 (0.98) |
