# Supplementary material for: Associations between the prevalence of influenza vaccination and patient’s knowledge about antibiotics: A cross-sectional study in the framework of the APRES-project in Austria
Source: BMC Public Health. 2015 Sep 29;15:981. doi: 10.1186/s12889-015-2297-x (PMC4587920; doi:10.1186/s12889-015-2297-x)
Supplement: Additional file 3: — Crude regression model for the association of the AB knowledge score with the likelihood to be vaccinated in both years surveyed (only “yes” and “no” answers regarding vaccination status were taken into account, “don’t know” answers were excluded). (DOCX 11 kb) [file 12889_2015_2297_MOESM3_ESM.docx]

Additional file 3. Crude regression model for the association of the AB knowledge score with the likelihood to be vaccinated in both years surveyed (only “yes” and “no” answers regarding vaccination status were taken into account, “don´t know” answers were excluded)

| Variable | Model (crude) | |
| --- | --- | --- |
|  | OR (CI 95%) | p |
| AB knowledge score | 1.21 (1.09-1.35) | <0.001 |
